# A Glimpse of Memory Through the Eyes: Pupillary Responses Measured During Encoding Reflect the Likelihood of Subsequent Memory Recall in an Auditory Free Recall Test

**DOI:** 10.1177/23312165221130581

**Published:** 2022-10-27

**Authors:** Andreea Micula, Jerker Rönnberg, Patrycja Książek, Reena Murmu Nielsen, Dorothea Wendt, Lorenz Fiedler, Elaine Hoi Ning Ng

**Affiliations:** 1 Department of Behavioural Sciences and Learning, Linnaeus Centre HEAD, Swedish Institute for Disability Research, 4566 Linköping University, Linköping, Sweden; 2 84686 Oticon A/S, Smørum, Denmark; 3 Amsterdam UMC, Vrije Universiteit Amsterdam, Otolaryngology-Head and Neck Surgery, Ear and Hearing, Amsterdam Public Health Research Institute, Amsterdam, The Netherlands; 4 Eriksholm Research Centre, Snekkersten, Denmark; 5 Hearing Systems, Hearing Systems Group, Department of Electrical Engineering, Technical University of Denmark, Kongens Lyngby, Denmark

**Keywords:** free recall, subsequent memory recall, pupillary responses, working memory

## Abstract

The aim of the current study was to investigate whether task-evoked pupillary responses measured during encoding, individual working memory capacity and noise reduction in hearing aids were associated with the likelihood of subsequently recalling an item in an auditory free recall test combined with pupillometry. Participants with mild to moderately severe symmetrical sensorineural hearing loss (n = 21) were included. The Sentence-final Word Identification and Recall (SWIR) test was administered in a background noise composed of sixteen talkers with noise reduction in hearing aids activated and deactivated. The task-evoked peak pupil dilation (PPD) was measured. The Reading Span (RS) test was used as a measure of individual working memory capacity. Larger PPD at a single trial level was significantly associated with higher likelihood of subsequently recalling a word, presumably reflecting the intensity of attention devoted during encoding. There was no clear evidence of a significant relationship between working memory capacity and subsequent memory recall, which may be attributed to the SWIR test and RS test being administered in different modalities, as well as differences in task characteristics. Noise reduction did not have a significant effect on subsequent memory recall. This may be due to the background noise not having a detrimental effect on attentional processing at the favorable signal-to-noise ratio levels at which the test was conducted.

## Introduction

Studying listening effort or cognitive resource allocation in both individuals with normal hearing and hearing impairment by combining pupillometry and free recall tasks is an increasingly popular method (Bönitz et al., 2021; Micula et al., 2020, 2021; Unsworth & Miller, 2020; Zekveld, Koelewijn, et al., 2018; Zekveld, Kramer, et al., 2018; 2018b; Zhang et al., 2021). Typically, such studies investigate the relationship between pupillary responses measured during a task and overall behavioral performance. However, this combination of measures offers the possibility to investigate cognitive resource allocation from different perspectives. For instance, some studies have explored whether the magnitude of task-evoked pupillary responses measured during encoding was associated with the likelihood of subsequently recalling heard items (Goldinger & Papesh, 2012; Kucewicz et al., 2018; Miller et al., 2019; Papesh et al., 2012), even at a single-trial level (Bergt et al., 2018). These studies were conducted on groups of participants with normal hearing. Therefore, the purpose of the current study was to investigate this in a group of hearing aid users. Since it has been shown that noise reduction in hearing aids and individual working memory capacity have an effect on auditory recall performance (Micula et al., 2020, 2021; Ng et al., 2013, 2015), the effect of these factors on the likelihood of subsequent memory recall was also of interest.

### Pupillary Responses as Indices of the Likelihood of Subsequent Memory Recall

Pupillary responses reflect the noradrenergic function of the locus coeruleus (Murphy et al., 2014). The magnitude of the task-evoked pupillary responses, which reflects transient changes in response to a stimulus, has been shown to index the allocation of attentional processing resources to a task (Aston-Jones & Cohen, 2005; Beatty, 1982; de Gee et al., 2017; Joshi & Gold, 2020; Kahneman, 1973; Laeng et al., 2012; Miller et al., 2019; Zekveld, Koelewijn, et al., 2018). Studies from the fields of cognitive psychology and neuroscience have demonstrated that the magnitude of task-evoked pupillary responses measured during item encoding reflects the likelihood of subsequent memory recall. Generally, increased pupillary responses at the time of encoding items into memory are associated with correct subsequent recognition or recall, reflecting a higher degree of attentional processing or allocation of resources to maintaining words in memory (Bergt et al., 2018; Kucewicz et al., 2018; Miller et al., 2019; Papesh et al., 2012).

According to Kucewicz et al. (2018), the likelihood of subsequent memory recall indexed by pupillary responses should be explored through additional memory tasks, in order to learn whether similar findings can be obtained using different paradigms or stimuli. This would provide information about whether the magnitude of task-evoked pupillary responses is a reliable index of attentional allocation for various tasks. In the current study, a combination of the Sentence-final Word Identification and Recall (SWIR) test (Ng et al., 2013, 2015) with pupillometry is used in order to investigate whether pupillary responses measured during encoding are associated with subsequent memory recall in a group of hearing aid users.

### Investigating Subsequent Memory Recall by Combining the Sentence-final Word Identification and Recall (SWIR) Test and Pupillometry

The SWIR test is an auditory recall test, which was originally designed to measure the effects of noise reduction in hearing aids and background noise on memory for heard speech (Ng et al., 2013, 2015). The task of the SWIR test consists of listening to lists of sentences, repeating the last word immediately after each sentence, and when the list is finished, recalling as many of the repeated words as possible. Recent studies have combined the SWIR test and pupillometry in order to investigate how noise reduction in hearing aids and manipulation of task difficulty affect cognitive resource allocation to speech recall in background noise (Bönitz et al., 2021; Micula et al., 2021). However, no previous studies have used the SWIR test to investigate which items are more likely to be recalled based on the magnitude of pupillary responses.

#### The Association Between Recall Performance on the SWIR Test and Working Memory Capacity

Recall performance in the SWIR test is closely linked to working memory capacity. It has been shown that there is a significant positive correlation between working memory capacity and recall performance (Micula et al., 2020; Ng et al., 2013). Individuals with higher working memory capacity presumably have the ability to exert a higher degree of task engagement or allocate more attentional processing resources to overcome challenges posed by difficult listening situations (Miller et al., 2019; Rönnberg et al., 2013, 2019). Consequently, working memory capacity and its relationship with subsequent memory recall is investigated in the present study.

#### The Association Between Noise Reduction in Hearing Aids and Recall Performance in the SWIR Test

Several studies have been conducted using the Swedish and the Danish versions of the SWIR test in order to investigate the effect of noise reduction on recall performance in individuals with hearing loss (Micula et al., 2020, 2021; Ng et al., 2013, 2015). These studies demonstrated that recall performance in the SWIR test was significantly better when noise reduction was activated compared to when it was not. Attenuating the background noise facilitates segregation of the target speech, thus freeing up resources that would otherwise be needed for speech processing to be used for storing speech in memory (Ng & Rönnberg, 2020; Rönnberg et al., 2013). Based on the evidence regarding the link between noise reduction and recall performance in the SWIR test, the effect of noise reduction on subsequent memory recall is investigated.

##### Controlling for Serial Position

The serial position effect (Murdock, 1974) was not taken into consideration in most studies investigating pupillary responses as an index of the likelihood of subsequent memory recall. This effect refers to the superior memory performance for items presented at the beginning (primacy) and end (recency) of a list, compared to those in the middle. Recall performance in the SWIR test tends to exhibit the typical U-shaped pattern in accordance with the serial position effect (Ng et al., 2013, 2015). Hence, the effect of serial position on subsequent memory recall is accounted for in the current study.

### Aims of the Study

The present study was conducted in order to investigate subsequent memory recall using measures relevant to the field of cognitive hearing science. We expand on previous findings by testing participants with hearing loss in background noise with hearing aid noise reduction on and off, which has not been under investigation in earlier studies. This is of interest since individuals with hearing loss need to allocate more resources to understand speech in background noise than individuals with normal hearing, even when speech is loud enough to be understood, which leaves fewer resource available for storing speech in memory (Lunner et al., 2009; Pichora-Fuller et al., 2016).

The first aim was to investigate whether the magnitude of the task-evoked peak pupil dilation (PPD) measured while listening to a SWIR test sentence and preparing to encode the target word into memory may be an index of the likelihood of subsequent memory recall. Based on previous findings, we hypothesize that the PPD will be larger for subsequently recalled words compared to those that are not recalled (Bergt et al., 2018; Kucewicz et al., 2018; Miller et al., 2019; Papesh et al., 2012).

The second aim was to investigate whether working memory capacity measured using the Reading Span (RS) test has an effect on the likelihood of subsequent memory recall. Previous studies have shown that individuals with higher working memory capacity perform better in the SWIR test (Micula et al., 2020; Ng et al., 2013). However, these studies focused on the relationship between working memory capacity and overall recall performance in the SWIR test rather than recall performance at a single-item level. It is expected that higher working memory capacity will be associated with higher likelihood of recalling a target word.

The third aim was to investigate whether noise reduction in hearing aids has an effect on the likelihood of subsequent memory recall at a single-trial level. Since recall performance in the SWIR test is generally better with noise reduction on (Micula et al., 2020, 2021; Ng et al., 2013, 2015) it is expected that the likelihood will be higher when noise reduction is on compared to off.

## Materials and Methods

### Participants

Twenty-five native Danish speakers with mild to moderately severe symmetrical sensorineural hearing loss were recruited from the database at Eriksholm Research Center, Snekkersten, Denmark. A subset of the collected data was used for the purpose of the current study. Four participants were excluded from the analyses, three due to the amount of missing pupillometry data (see Pupillometry) and one for not having completed the RS test. The mean age of the 21 included participants (8 female, 13 male) was 58 years (SD = 11.3, range: 22–73). It should be noted that the large age range is driven by a single participant, as the age gap between the two youngest participants is of 20 years. The four-frequency pure tone average (PTA) at 0.5, 1, 2 and 4 kHz was 49.2 dB HL (SD = 11.5) for the left ear and 49.3 dB HL (SD = 12.5) for the right ear. All participants were experienced hearing aid users and had normal or corrected to normal vision, as well as no history of eye surgery or eye disease. The study was exempted from ethical application by the Science Ethics Committee for the Capital Region of Denmark (journal no. H-20028542). All participants signed a written informed consent form, and the study was conducted in accordance with the Declaration of Helsinki.

### Assessment Tools

#### The Sentence-final Word Identification and Recall (SWIR) Test

A Danish version of the SWIR test was used in the current study (Lunner et al., 2016). This version is composed of sentences from the Danish Hearing In Noise Test (HINT) (Nielsen & Dau, 2011). The SWIR test material was expanded compared to the one used by Lunner et al. (2016) by re-combining the sentences into new lists. The participants were asked to listen to lists of seven sentences and repeat the last word immediately after each sentence. After the last sentence there was a silent interval of four seconds. This was followed by a beep tone cueing the free recall task, during which participants were asked to recall as many of the repeated target words as possible in any order. It should be noted that misheard words were accepted if they were correctly recalled.

#### Pupillometry

Pupillary responses were measured using the Tobii Spectrum Eye Tracker (Tobii Technology AB 2019) at a sampling frequency of 1200 Hz. Data from the right eye of each participant was analyzed per default, unless more data was available for the left eye. A 1s-long sliding window (in total 1200 samples) was used for detecting blinks. Samples with pupil dilation below the minimum threshold, set to 3 SDs below the mean pupil size within the sliding window, were considered to be blinks. These were removed from the signal, including 77 samples (64ms) before and the 181 samples (151ms) after (Siegle et al., 2008). Linear interpolation was used to replace the missing values after blink removal. In the present study, a trial consists of a single sentence. The trials with less than 60% of valid data were discarded. Furthermore, participants for whom more than 15% of the sentences were missing were excluded from the analyses. Three participants were excluded based on these criteria. For the remaining participants, 1.65% of the trials were discarded on average. Thus, a mean of 192.76 valid trials remained per participant out of a total of 196 trials. On average, 9.92% of the valid trial data was interpolated.

**Figure 1.**
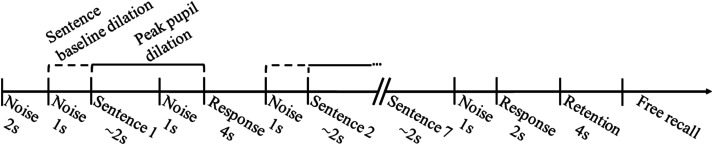
Illustration of the time course of a SWIR test list including the time intervals during which the sentence baseline dilation (dotted line) and peak pupil dilation (continuous line) were calculated.

The PPD during encoding was calculated as the maximum pupil dilation that occurred in a time interval of four seconds starting at each sentence onset. Additionally, the PPD of each sentence was corrected for its corresponding baseline dilation. The PPD was calculated in the same way in previous studies combining the SWIR test and pupillometry (Bönitz et al., 2021; Micula et al., 2021). The HINT material, which the SWIR test is composed of, has been thoroughly validated in order to ensure that none of the sentences elicit especially strong feelings, leading to higher levels of arousal (Nielsen & Dau, 2011). However, it cannot be excluded that individual differences in arousal may arise as a result of the content of various sentences. By calculating the PPD in relation to the sentence baseline, the effect of individual differences in arousal on the task-evoked pupillary responses is minimized. Figure 1 illustrates the time intervals during which the pupillary responses were recorded over the course of the SWIR test.

#### Reading Span (RS) Test

The RS test is a suitable measure of working memory capacity, since it requires simultaneous processing and storage of information (Daneman & Carpenter, 1980; Rönnberg et al., 1989). A Danish version of the test was used (Petersen et al., 2016), but it was modified based on the short version of the Swedish RS test (Ng et al., 2013, 2015; Rönnberg et al., 1989). The three-word sentences were arranged in lists of three-, four- and five sentences. Additionally, one list of three sentences was administered for procedural training. Two lists of each length (24 sentences in total) were presented in ascending order on a laptop screen. Half of the sentences were sensible, and half were absurd. The participants were instructed to read the sentences and verbally indicate after each one whether it was sensible or absurd. The participants were asked to either recall the first or the last words of each sentence at the end of each list. The RS test score was obtained by calculating the percentage of correctly recalled words, irrespective of order (Petersen et al., 2016).

### Test Conditions and Set-up

The test participants were seated in a soundproof anechoic chamber. The SWIR test sentences were presented in a background noise composed of 16-talker babble. The level of the background noise was fixed at 70 dB SPL, while level of the target sentences was individualized for each participant to a signal-to-noise ratio (SNR) estimated to result in 95% word recognition (see Procedure). The background noise began three seconds prior the first sentence of the SWIR test list and one second prior to the remaining six sentences in the list. The background noise stopped one second after the end of each sentence (Figure 1). The target sentences were presented from a frontal loudspeaker placed at 0°. The background noise was presented from four loudspeakers placed at ±112.5° and ±157.5°. Each of these loudspeakers played recordings of two male and two female native Danish speakers reading different passages of a newspaper article. All loudspeakers were placed at a distance of 1.2 m from the participant. The eye-tracker was placed in between the frontal loudspeaker and the participant at a distance of 60 cm. The participants were instructed to fixate their gaze on a black cross shown at the center of a screen on gray background, thereby ensuring constant luminance.

The participants received appropriate amplification via hearing aids (Oticon OpnS1^TM^ and Oticon More^TM^ mini-Receiver-in-the-ear) based on their individual audiometric thresholds. For half of the SWIR test lists the noise reduction in the hearing aids was activated and for the other half it was not.

### Procedure

The test participants took part in one test session, during which the SWIR test was administered first, followed by the RS test. In order to estimate the SNR required for the SWIR test, the HINT was administered using a modified procedure in order to achieve 80% speech intelligibility with noise reduction off. At the beginning of the test, both the target sentences and the babble were presented at 70 dB SPL, after which the level of the target speech fluctuated based on the participants’ responses. The SNR was decreased by 0.8 dB if a sentence was correctly repeated, otherwise the SNR was increased by 3.2 dB. For the first five sentences, however, the step size was twice as large. The SNR resulting from the HINT test was used as a starting point for the SWIR test training. Four lists were administered with noise reduction off for procedural training, as well as for making SNR adjustments if needed in order to achieve 95% word recognition in the SWIR test (Lunner et al., 2016; Micula et al., 2020, 2021). The SNR obtained from the HINT was not modified if six or seven (86–100%) last words from the SWIR test training list were repeated correctly. If four or five words were repeated correctly, the SNR was increased by 1 dB and if zero to three words were repeated correctly, the SNR was increased by 2 dB. The SWIR test was conducted using the SNR obtained after the fourth training list. The mean SNR in the current study was 6.6 dB (SD = 3.2), which is close to the level reported by Micula et al. (2021). In total, 28 SWIR test lists of seven sentences were administered, half with noise reduction on and half with noise reduction off. At the end of the test session, the RS test was completed.

### Statistical Analysis

A priori power analyses were conducted in G*Power in order to ensure a sufficiently large sample size. The information provided in previous studies that have investigated the effects of noise reduction on recall performance in the SWIR test in groups of participants with hearing loss was used. Micula et al. (2021) report an effect size of ɳ^2^ = 0.59 for the main effect of noise reduction on recall performance. Setting the alpha error probability to 0.05, the power to 0.80 and given that the number of measurements is two (noise reduction on vs. off), the estimated total sample size is four. The SWIR test has been shown to be a robust tool for capturing the effect of noise reduction on recall performance (Micula et al., 2020; Ng et al., 2013, 2015). These studies have used the same amount or fewer SWIR test lists per condition compared to the present study. For the link between PPD and subsequent memory recall, the information provided by Bergt et al. (2018) was used. Given an effect size of ɳ^2^ = 0.10, the number of measurements being two, setting the alpha error probability to 0.05 and the power to 0.80, the estimated sample size is 20. This requirement was met with data from 21 participants.

A binary logistic mixed effects model was constructed using the glmer() function from the lme4 package (Bates et al., 2015, 2021) in R (R Core Team, 2020). The analysis used a generalized linear mixed model fit by maximum likelihood (Laplace approximation) and logit link function, using the optimizer “bobyqa”. This analysis was most suitable due to the repeated-measures design and the binary outcome. It should be noted that only the target words that were repeated were included in the analysis. Only 2% of the target words were not heard amongst all participants.

Subsequent memory recall (recalled vs. not recalled) was set as the dependent variable. PPD, RS test score, age and PTA were defined as continuous variables and were centered by subtracting the mean. Noise reduction was defined as a categorical variable (on vs. off). These variables were included in the model as fixed effects. PTA and age were included, since the target group includes individuals with hearing loss of varying ages. Test participant and trial were added as crossed random factors, in order to account for the primacy and recency effects.

## Results

The mean RS test score was 41.8% (SD = 12.1), which is similar to the scores reported by Ng et al. (2013, 2015). The mean recall performance in the SWIR test was 58.0% (SD = 18.3), indicating that the data is relatively balanced in terms of recalled and not recalled words. The level of the recall performance is very close to the recall performance reported by Micula et al. (2020, 2021).

Table 1 presents the output of the binary logistic mixed effects model. A likelihood ratio test showed that the fit of the model was significantly better than the null model, χ^2^(5) = 12.12, *p* = .033. The variance inflation factor for each variable was calculated. All values were very close to 1, demonstrating that multicollinearity between variables was low. Additionally, the conditional R^2^ indicated that the fixed and random factors explained 37.7% of the variance, while the marginal R^2^ indicated that the fixed effects alone accounted for 2.6% of the variance (Nakagawa & Schielzeth, 2013).

**Table 1.** Output Summary of the Binary Logistic Mixed Effects Model.

	*β* (95% CI)	Odds ratio *β* (95% CI)	*p*
Intercept	0.61 (−0.33, 1.56)	1.85 (0.72, 4.76)	.20
PPD	0.60 (0.13, 1.07)	1.83 (1.14, 2.91)	.01*
RS test score	0.03 (0.00, 0.05)	1.03 (1.00, 1.05)	.04*
Noise reduction	−0.01 (−0.15, 0.13)	0.99 (0.86, 1.14)	.92
Age	−0.01 (−0.03, 0.02)	0.99 (0.97, 1.02)	.61
PTA	0.01 (−0.02, 0.03)	1.01 (0.98, 1.03)	.53

The odds ratio was obtained by exponentiating the β coefficient. CI, confidence interval. *p < .05.

An ANOVA (Type II Wald χ^2^ tests) was conducted on the model, which showed a significant main effect of PPD, χ^2^(1) = 6.37, *p* = .011, as well as a significant main effect of RS test score, χ^2^(1) = 4.13, *p* = .042, demonstrating that these variables were significantly associated with the likelihood of subsequent memory recall. However, the effect of noise reduction, χ^2^(1) = 0.01, *p* = .92, was not significant, indicating that noise reduction did not have an effect on the likelihood of subsequent memory recall. The effects age, χ^2^(1) = 0.27, *p* = .61, and PTA, χ^2^(1) = 0.39, *p* = .53 were not significant either. A figure of the model plots is included in the supplementary materials showing the predicted associations between each fixed effect and the likelihood of subsequent memory recall (Supplementary Figure 1).

**Figure 2.**
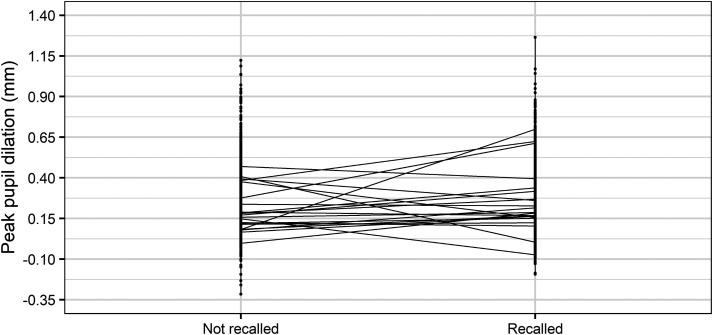
Comparison of individual peak pupil dilation values (dots) and individual mean peak pupil dilation values (sloping lines) for recalled compared to not recalled SWIR test target words.

The results demonstrated that a larger PPD during encoding was associated with a higher probability of subsequent memory recall. This is in line with the prediction made in the first hypothesis. Figure 2 shows the individual PPD values for recalled and not recalled words (dots). The sloping lines show the difference between individual mean PPD values for recalled and not recalled words.

**Figure 3.**
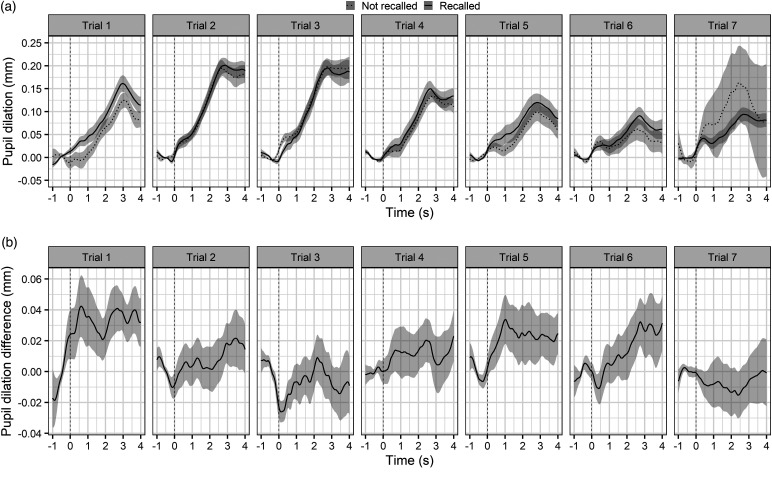
(a) Time course of the pupillary dilation averaged per trial for recalled (solid line) and not recalled (dotted line) words. (b) Difference in the time course of the pupillary dilation between recalled and not recalled words averaged per trial. The shaded areas show the standard error.

Figure 3 shows the time course of the pupillary dilation for words that were recalled (solid line) and words that were not recalled (dotted line) averaged for each of the seven trials (a), as well as the difference between the time course of the responses for recalled and not recalled words (b). On average, pupillary responses for words that are subsequently recalled are generally larger. It should be noted that the reversed pattern and large standard error in the last trial may be due to the small amount of data for not recalled words. Only 3.8% of the target items in the last trial were not recalled. This can be attributed to a strong recency effect on recall performance and is accounted for by including trial as a random factor.

Based on the studies that the current paper builds upon (Bergt et al., 2018; Kucewicz et al., 2018; Miller et al., 2019; Papesh et al., 2012), the hypothesis focused on a linear relationship between PPD and the likelihood of subsequent memory recall. However, the possibility of a non-linear relationship may be considered (de Gee et al., 2017; Van Kempen et al., 2019). An exploratory analysis was conducted by including a quadratic term for the PPD in the binary logistic mixed effects model. Both the linear and the quadratic relationships between PPD and subsequent memory recall were significant. This finding suggests that the likelihood of subsequent memory recall increases with increasing PPD until a certain point, after which it decreases. The outcomes of this exploratory analysis are included in the supplementary materials (Supplementary Figure 2 and Supplementary Table).

**Figure 4.**
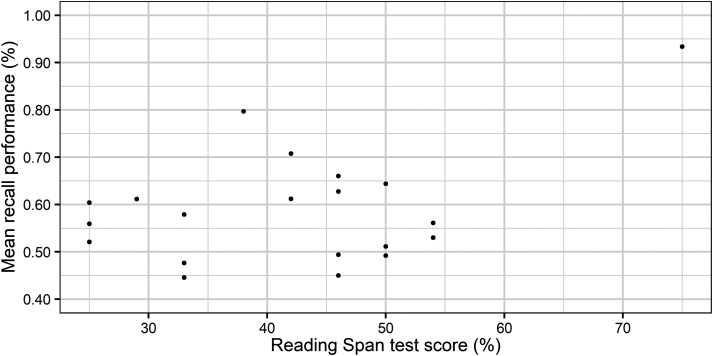
Mean recall performance as a function of individual reading span test score (dots).

Furthermore, the results showed that the higher the RS test score, the higher the probability of subsequent memory recall. However, one participant obtained a noticeably higher score in the RS test than the other participants. Figure 4 illustrates the mean recall performance in the SWIR test as a function of individual RS test score.

In order to investigate whether the significant relationship between RS test score and the likelihood of subsequent memory recall was driven by the highest RS test score, the statistical analysis was repeated after excluding the data of the respective participant. The results of the revised model indicate that the main effect of RS test score is not significant, χ^2^(1) = 0.02, *p* = 0.89. The remaining outcomes did not change in comparison to the original analysis.

**Figure 5.**
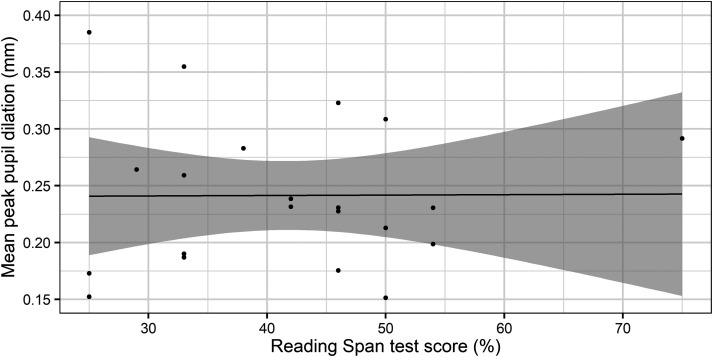
Correlation between peak pupil dilation and Reading Span test score. The black dots show the individual data points and the black line shows the regression slope. The shaded area shows the confidence interval at the 95%-level.

A post hoc Pearson's correlation analysis between PPD and RS test score was conducted using the function cor.test(). For this analysis, all PPD values were averaged per participant. The outcome of the correlation analysis was not significant, r = 0.007, *p* = .98. The relationship between PPD and RS test score is illustrated in Figure 5, demonstrating that there is no linear relationship between them.

## Discussion

The present study investigated whether the magnitude of the task-evoked PPD, working memory capacity measured using the RS test and noise reduction in hearing aids are linked to the likelihood of subsequent memory recall in the SWIR test.

The results showed a significant positive relationship between the magnitude of the PPD measured during encoding and subsequent memory recall. The larger the PPD when listening to a SWIR test sentence, the higher the likelihood of subsequently recalling the corresponding target word. This is in line with previous studies that have reported similar findings on task-evoked pupillary responses (Bergt et al., 2018; Kucewicz et al., 2018; Miller et al., 2019; Papesh et al., 2012). Papesh et al. (2012) obtained similar outcomes using an auditory word recognition accuracy test rather than a free recall test. The outcomes showed that task-evoked pupillary responses measured during encoding were larger for items that were correctly recognized compared to those that were not. Miller et al. (2019) and Kucewicz et al. (2018) have used free recall tasks composed of visually presented words, while Bergt et al. (2018) have used a free recall task composed of words presented auditorily. Free recall tasks are considered to give a better insight into search and retrieval from memory in comparison to memory-based multiple choice recognition tests (Bergt et al., 2018; Kucewicz et al., 2018). These three studies using free recall tasks also found that task-evoked pupillary responses measured during encoding are larger for words that are subsequently recalled compared to those that are not. Most studies have averaged pupillary responses across several trials. The present study alongside the study by Bergt et al. (2018) demonstrate that pupillary responses measured on a trial-by-trial basis are indices of the likelihood of subsequent memory recall.

The link between the likelihood of subsequent memory recall and task-evoked pupillary responses measured during encoding mainly stems from the association between pupil dilation and the noradrenergic function of the locus coeruleus. Since the noradrenergic function plays a crucial role in memory formation, task-evoked pupillary responses may provide a window into such processes (Bergt et al., 2018; Miller et al., 2019). Previous studies have associated task-evoked pupillary responses to encoding strength or the amount of cognitive resources allocated during encoding (Goldinger & Papesh, 2012; Kucewicz et al., 2018; Papesh et al., 2012). Miller et al. (2019) interpret the magnitude of task-evoked pupillary responses as an index of the intensity of attention devoted to items during encoding into long-term memory. The amount of attentional processing devoted to an item influences the strength of its memory representation. The greater the representation strength of an item, the higher the probability that it will be subsequently recalled (Miller et al., 2019; Rohrer, 1996). Based on the study by Miller et al. (2019), we speculated that the increased PPD during encoding of target words that are subsequently recalled in the SWIR test free recall phase reflects a higher intensity of attention devoted to those words. The exploratory analysis of a non-linear relationship between PPD and subsequent memory recall showed that an increase in PPD is associated with higher likelihood of recalling an item until a certain point. After that point a higher PPD was associated with a lower likelihood of subsequent memory recall. An explanation for this may be that the PPD values in the highest range reflect an increase in cognitive resources being allocated to correctly identifying an item, leaving fewer resources available for encoding (Lunner et al., 2009; Rönnberg et al., 2013). While this was not within the scope of the current paper, there are methods that allow disentangling the effect of cognitive resource allocation to listening and encoding on the pupillary response (Książek et al., under review).

The previous studies mentioned thus far included young healthy adults with no reported hearing loss and the testing was not conducted in background noise. Our study further builds on the evidence that task-evoked pupillary responses are reliable indices of subsequent memory recall by using a different test paradigm and a different test participant group. In the present study, participants with hearing loss completed the test in a background noise composed of 16-talker babble. When the quality of auditory information is low due to background noise and/or hearing loss, more attentional resources are needed to process the target speech (Pichora-Fuller et al., 2016; Rönnberg et al., 2013, 2019). Furthermore, background noise composed of speech babble might compete with the target speech for attentional resources due to its lexical-semantic content (Mattys et al., 2009; Ng & Rönnberg, 2019). Thus, our findings demonstrate that even in adverse listening conditions, the PPD measured during encoding is still a reliable index of subsequent memory recall on a trial-by-trial basis.

The findings of the initial analysis also indicate that working memory capacity has an effect on subsequent memory recall. However, the significant relationship may be driven by the data from a single participant, who scored much higher on the RS test than the rest of the participants (Figure 4). After removing the data of the respective participant, the relationship between working memory capacity and subsequent memory recall was no longer significant. The outcome of the initial analysis was expected since the correlation between working memory capacity and recall performance in the SWIR test has been well-established (Micula et al., 2020; Ng et al., 2013). Miller et al. (2019) suggest that individuals with higher working memory capacity are able to devote more attentional processing during encoding. It is important to note that in comparison to previous studies, it is not the overall recall performance that is taken into account in the present study, but the likelihood of subsequent memory recall of each individual target word. Although the lack of a significant relationship between working memory capacity and subsequent memory recall that resulted from the revised model was unexpected, several factors may account for it. First, measures presented in the auditory modality may create a disadvantage for individuals with hearing impairment compared to measures presented in the visual modality (Cacace & McFarland, 2013; Smith & Pichora-Fuller, 2015). Second, differences in speech materials (sensible and absurd sentences in the RS test vs. only sensible sentences in the SWIR test) and task characteristics (repetition of first or last word in the RS test vs. repetition of the last word in the SWIR test) may have an effect on the relationship between the measures (Smith & Pichora-Fuller, 2015).

Given that PPD may be interpreted as an index of the intensity of attentional processing, and individuals with higher working memory capacity are expected to be able to allocate more attentional processing resources during encoding, it could be assumed that these variables are closely related. However, there does not seem to be a linear relationship between them (Figure 5). The PPD is a dynamic measure of the amount of attentional processing resources spent during a particular trial, while RS test score yields a single value reflecting the maximum individual working memory capacity. Thus, pupillary responses may be more sensitive to temporal fluctuations in attentional processing than the RS test score. Furthermore, Baddeley's working memory model assumes that this system contains multiple components, which may be reflected in the RS test score. Additionally, pupillary responses have been shown to be affected by factors beyond cognitive function, such as arousal or emotion (Mathôt, 2018). Consequently, the lack of a significant correlation may be attributed to the PPD and RS test score reflecting different aspects of attentional processing, or even other cognitive or physiological mechanisms.

Despite the evidence from previous studies demonstrating that recall performance in the SWIR test is better with noise reduction on compared to off (Micula et al., 2020, 2021; Ng et al., 2013, 2015), the effect of noise reduction on subsequent memory recall was not significant. Previous studies have administered the SWIR test in a background noise composed of four talkers, which competes with the target talker for attentional resources due to lexical interference. The benefit of 16-talker babble is that it entails ecological plausibility, and it reduces the opportunities for glimpsing the target speech (Rosen et al., 2013). However, the latter attribute may not be relevant at the favorable SNR levels at which the SWIR test is administered. Additionally, the lexical interference may decrease as the competing speech becomes less intelligible with increasing number of talkers (Rosen et al., 2013). Micula et al. (2020) and Ng et al. (2013) showed that, unlike four-talker babble, speech-shaped noise did not disrupt recall performance when noise reduction was off. In the present study, 16-talker babble may have been perceived more similarly to speech-shaped noise, decreasing the need for noise reduction. Other studies have also failed to capture an effect of noise reduction on behavioral performance (Luts et al., 2010; Neher et al., 2014). Neher et al. (2014) did not find a significant effect of noise reduction on performance on a dual task administered in background noise recorded in a cafeteria, which only contained occasional portions of intelligible speech. Luts et al. (2010) likewise found that speech reception thresholds in multi-talker babble with different degrees of reverberation did not improve using various noise reduction algorithms. The authors of both studies point out that the lack of improvement in behavioral performance may be caused by the distortions introduced by the noise reduction algorithms.

It was not within the scope of this study to investigate the effects of age and PTA on subsequent memory recall. However, these variables were included in the analysis as they represent the characteristics that set the group of participants apart from those of the previous studies investigating subsequent memory recall. Neither age nor PTA had a significant effect on subsequent memory recall. This may be due to the age range of the participants being relatively homogenous, with the exception of one younger participant. Furthermore, the test participants received appropriate amplification and the SNR level was individualized to result in nearly perfect speech recognition.

## Conclusions

The findings of the present study demonstrate that the task-evoked PPD measured during encoding is a reliable index of the likelihood of subsequent memory recall on a trial-by-trial basis in the SWIR test. The PPD measured during encoding of words that were subsequently recalled was larger compared to words that are not recalled. Thus, PPD is considered to reflect the intensity of attention allocated to each target word during encoding. This corroborates previous findings (Bergt et al., 2018; Kucewicz et al., 2018; Miller et al., 2019; Papesh et al., 2012), but also generalizes the findings to participants with hearing impairment when listening to speech in background noise.

There was no clear evidence of a significant relationship between working memory capacity measured via the RS test and the likelihood of subsequent memory recall. The disadvantage posed by the auditorily administered SWIR test for participants with hearing impairment compared to the visually administered RS test or the differences related to the task characteristics may account for this. There was no effect of noise reduction on subsequent memory recall. This may be due to the 16-talker babble causing a low degree of lexical interference, thus being less detrimental for attentional processing than a babble noise composed of fewer talkers, especially at the favorable SNR levels at which the SWIR test is administered.

In conclusion, the findings of the present study show that the SWIR test combined with pupillometry can be used to obtain insights about which words heard in background noise are more likely to be subsequently recalled and which factors may affect this.

## Supplemental Material

sj-tiff-1-tia-10.1177_23312165221130581 - Supplemental material for A Glimpse of Memory Through the Eyes: Pupillary Responses Measured During Encoding Reflect the Likelihood of Subsequent Memory Recall in an Auditory Free Recall Test Click here for additional data file. Supplemental material, sj-tiff-1-tia-10.1177_23312165221130581 for A Glimpse of Memory Through the Eyes: Pupillary Responses Measured During Encoding Reflect the Likelihood of Subsequent Memory Recall in an Auditory Free Recall Test by Andreea Micula, Jerker Rönnberg, Patrycja Książek, Reena Murmu Nielsen, Dorothea Wendt, Lorenz Fiedler and Elaine Hoi Ning Ng in Trends in Hearing

sj-docx-2-tia-10.1177_23312165221130581 - Supplemental material for A Glimpse of Memory Through the Eyes: Pupillary Responses Measured During Encoding Reflect the Likelihood of Subsequent Memory Recall in an Auditory Free Recall Test Click here for additional data file. Supplemental material, sj-docx-2-tia-10.1177_23312165221130581 for A Glimpse of Memory Through the Eyes: Pupillary Responses Measured During Encoding Reflect the Likelihood of Subsequent Memory Recall in an Auditory Free Recall Test by Andreea Micula, Jerker Rönnberg, Patrycja Książek, Reena Murmu Nielsen, Dorothea Wendt, Lorenz Fiedler and Elaine Hoi Ning Ng in Trends in Hearing

sj-tiff-3-tia-10.1177_23312165221130581 - Supplemental material for A Glimpse of Memory Through the Eyes: Pupillary Responses Measured During Encoding Reflect the Likelihood of Subsequent Memory Recall in an Auditory Free Recall Test Click here for additional data file. Supplemental material, sj-tiff-3-tia-10.1177_23312165221130581 for A Glimpse of Memory Through the Eyes: Pupillary Responses Measured During Encoding Reflect the Likelihood of Subsequent Memory Recall in an Auditory Free Recall Test by Andreea Micula, Jerker Rönnberg, Patrycja Książek, Reena Murmu Nielsen, Dorothea Wendt, Lorenz Fiedler and Elaine Hoi Ning Ng in Trends in Hearing

sj-docx-4-tia-10.1177_23312165221130581 - Supplemental material for A Glimpse of Memory Through the Eyes: Pupillary Responses Measured During Encoding Reflect the Likelihood of Subsequent Memory Recall in an Auditory Free Recall Test Click here for additional data file. Supplemental material, sj-docx-4-tia-10.1177_23312165221130581 for A Glimpse of Memory Through the Eyes: Pupillary Responses Measured During Encoding Reflect the Likelihood of Subsequent Memory Recall in an Auditory Free Recall Test by Andreea Micula, Jerker Rönnberg, Patrycja Książek, Reena Murmu Nielsen, Dorothea Wendt, Lorenz Fiedler and Elaine Hoi Ning Ng in Trends in Hearing

sj-docx-5-tia-10.1177_23312165221130581 - Supplemental material for A Glimpse of Memory Through the Eyes: Pupillary Responses Measured During Encoding Reflect the Likelihood of Subsequent Memory Recall in an Auditory Free Recall Test Click here for additional data file. Supplemental material, sj-docx-5-tia-10.1177_23312165221130581 for A Glimpse of Memory Through the Eyes: Pupillary Responses Measured During Encoding Reflect the Likelihood of Subsequent Memory Recall in an Auditory Free Recall Test by Andreea Micula, Jerker Rönnberg, Patrycja Książek, Reena Murmu Nielsen, Dorothea Wendt, Lorenz Fiedler and Elaine Hoi Ning Ng in Trends in Hearing
