# Supplementary figures and images for: A Glimpse of Memory Through the Eyes: Pupillary Responses Measured During Encoding Reflect the Likelihood of Subsequent Memory Recall in an Auditory Free Recall Test
Source: Trends Hear. 2022 Oct 27;26:23312165221130581. doi: 10.1177/23312165221130581 (PMC9620000; doi:10.1177/23312165221130581)

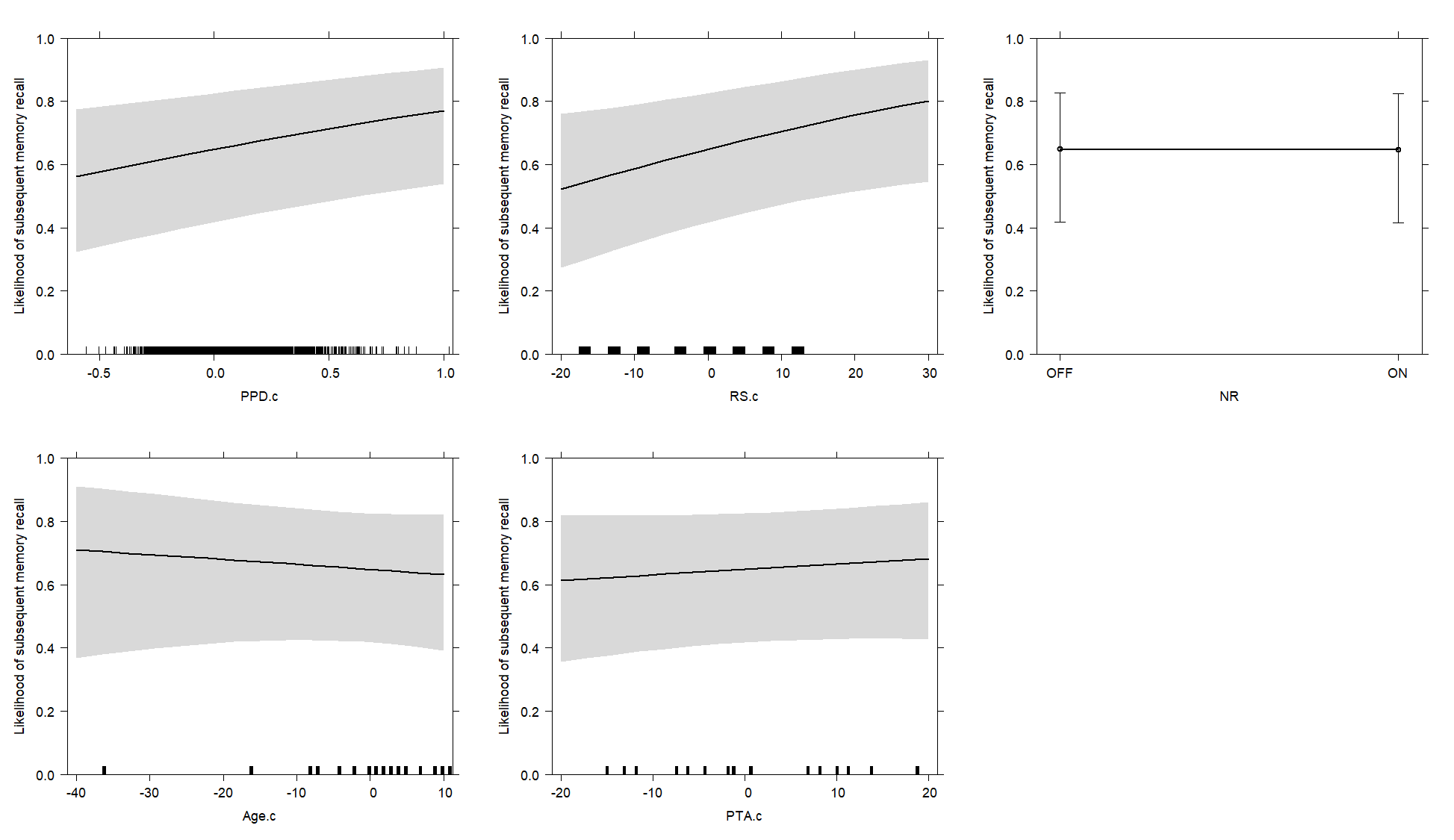

Supplement: sj-tiff-1-tia-10.1177_23312165221130581 - Supplemental material for A Glimpse of Memory Through the Eyes: Pupillary Responses Measured During Encoding Reflect the Likelihood of Subsequent Memory Recall in an Auditory Free Recall Test [file sj-tiff-1-tia-10.1177_23312165221130581.tiff]

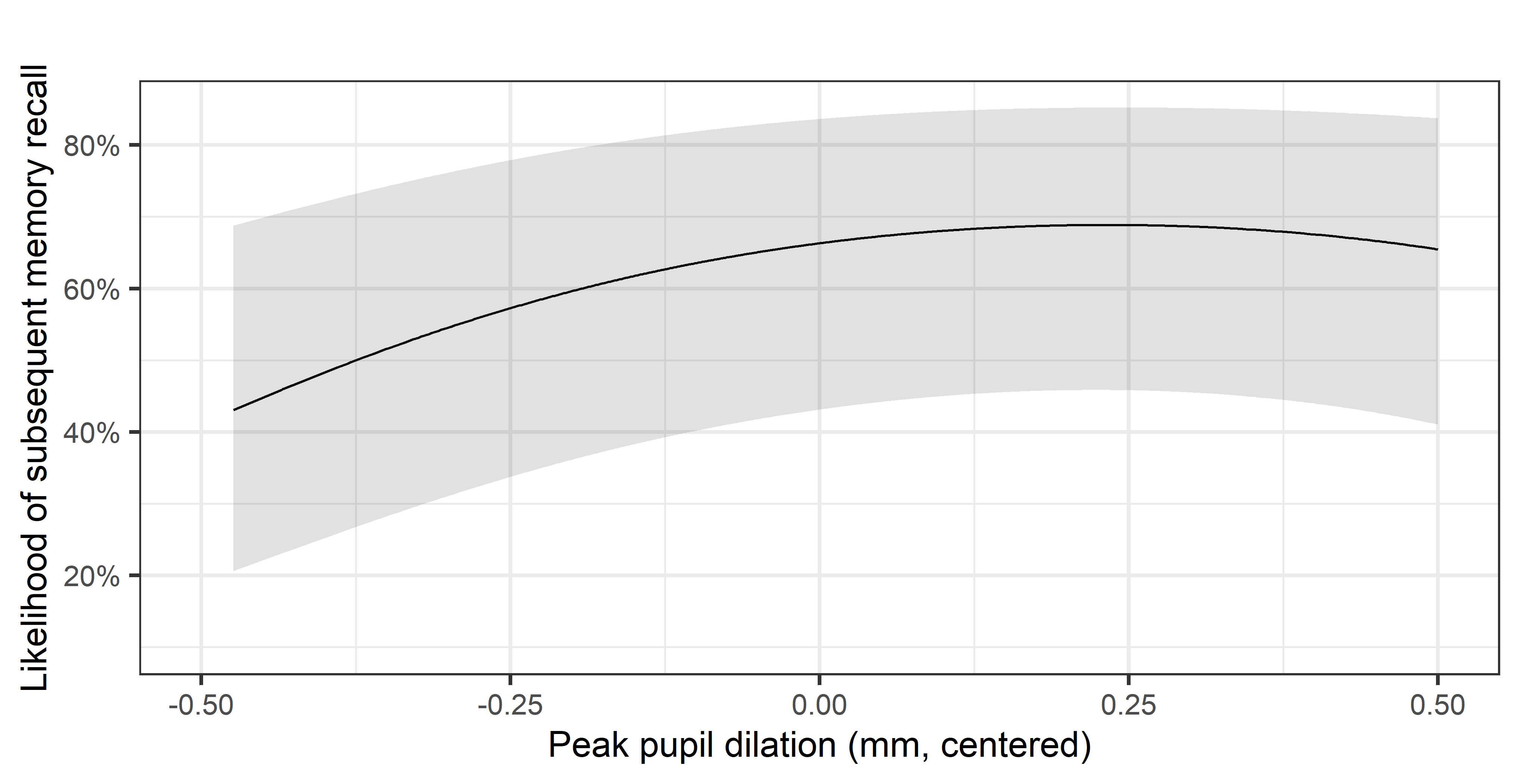

Supplement: sj-tiff-3-tia-10.1177_23312165221130581 - Supplemental material for A Glimpse of Memory Through the Eyes: Pupillary Responses Measured During Encoding Reflect the Likelihood of Subsequent Memory Recall in an Auditory Free Recall Test [file sj-tiff-3-tia-10.1177_23312165221130581.tiff]
