# Supplementary material for: A Glimpse of Memory Through the Eyes: Pupillary Responses Measured During Encoding Reflect the Likelihood of Subsequent Memory Recall in an Auditory Free Recall Test
Source: Trends Hear. 2022 Oct 27;26:23312165221130581. doi: 10.1177/23312165221130581 (PMC9620000; doi:10.1177/23312165221130581)
Supplement: sj-docx-2-tia-10.1177_23312165221130581 - Supplemental material for A Glimpse of Memory Through the Eyes: Pupillary Responses Measured During Encoding Reflect the Likelihood of Subsequent Memory Recall in an Auditory Free Recall Test [file sj-docx-2-tia-10.1177_23312165221130581.docx]

Supplementary figure 1: Model plots showing the predicted partial slopes between each fixed effect (x-axis) and the likelihood of subsequent memory recall (y-axis). The continuous fixed effects are centered. The individual data points are shown above the x-axis for the continuous fixed effects. The shaded areas and error bars show the confidence interval at the 95%-level.
