# Supplementary material for: A Glimpse of Memory Through the Eyes: Pupillary Responses Measured During Encoding Reflect the Likelihood of Subsequent Memory Recall in an Auditory Free Recall Test
Source: Trends Hear. 2022 Oct 27;26:23312165221130581. doi: 10.1177/23312165221130581 (PMC9620000; doi:10.1177/23312165221130581)
Supplement: sj-docx-4-tia-10.1177_23312165221130581 - Supplemental material for A Glimpse of Memory Through the Eyes: Pupillary Responses Measured During Encoding Reflect the Likelihood of Subsequent Memory Recall in an Auditory Free Recall Test [file sj-docx-4-tia-10.1177_23312165221130581.docx]

Supplementary figure 2: Predicted quadratic relationship between the likelihood of subsequent memory recall based on the peak pupil dilation (PPD) magnitude when other variables are held fixed. The shaded area shows the confidence interval at the 95%-level. The PPD values are centered.
