# Supplementary material for: A Glimpse of Memory Through the Eyes: Pupillary Responses Measured During Encoding Reflect the Likelihood of Subsequent Memory Recall in an Auditory Free Recall Test
Source: Trends Hear. 2022 Oct 27;26:23312165221130581. doi: 10.1177/23312165221130581 (PMC9620000; doi:10.1177/23312165221130581)
Supplement: sj-docx-5-tia-10.1177_23312165221130581 - Supplemental material for A Glimpse of Memory Through the Eyes: Pupillary Responses Measured During Encoding Reflect the Likelihood of Subsequent Memory Recall in an Auditory Free Recall Test [file sj-docx-5-tia-10.1177_23312165221130581.docx]

**Supplementary table. Output summary of the exploratory binary logistic mixed effects model including a quadratic term for peak pupil dilation.**

|  | ***β* (95% CI)** | **Odds ratio *β* (95% CI)** | ***p*** |
| --- | --- | --- | --- |
| **Intercept** | 0.61 (-0.34, 1.57) | 1.85 (0.71, 4.79) | .21 |
| **PPD** | 6.60 (1.57, 11.64) | 736.61 (4.78, 113398.13) | .01* |
| **PPD^2^** | -6.90 (-11.81, -1.98) | 0.00 (0.00, 0.14) | .01* |
| **RS test score** | 0.03 (0.00, 0.05) | 1.03 (1.00, 1.05) | .05* |
| **Noise reduction** | -0.01 (-0.15, 0.13) | 0.99 (0.86, 1.14) | .91 |
| **Age** | -0.01 (-0.03, 0.02) | 0.99 (0.97, 1.02) | .57 |
| **PTA** | 0.01 (-0.02, 0.03) | 1.01 (0.98, 1.03) | .49 |

*The odds ratio was obtained by exponentiating the β coefficient. CI, confidence interval. *p<.05*
